# Computed tomography evaluation of pancreatic steatosis: correlation with COVID-19 prognosis

**DOI:** 10.2217/fvl-2021-0257

**Published:** 2021-02-10

**Authors:** Serkan Guneyli, Hakan Dogan, Omer Tarik Esengur, Hur Hassoy

**Affiliations:** ^1^Department of Radiology, Bakircay University School of Medicine, Izmir, 35665, Turkey; ^2^Department of Radiology, Koc University School of Medicine, Istanbul, 34010, Turkey; ^3^Koc University School of Medicine, Istanbul, 34010, Turkey; ^4^Department of Public Health, Ege University School of Medicine, Izmir, 35040, Turkey

**Keywords:** chest, computed tomography, COVID-19, pancreas, steatosis

## Abstract

**Aim:** To investigate the relationship between pancreatic steatosis (PS) assessed on computed tomography (CT) and COVID-19 prognosis. **Materials & methods:** This retrospective study covers 396 patients with COVID-19 (mean age: 52.50 ± 16.86 years), who underwent unenhanced chest CT. PS was compared with clinical findings, comorbidities, requirements for hospitalization, intubation and intensive care unit (ICU), length of hospitalization and death. **Results:** PS was found to be strongly correlated with the severity of clinical findings and hospitalization rates (p < 0.001). In hospitalized patients, length of hospitalization (p = 0.002) and rates of ICU requirement (p = 0.003) were higher in patients with PS. **Conclusion:** PS, correlated with clinical severity and hospitalization requirement, is an independent risk factor for COVID-19.

## Plain language summary

Fat accumulation in the pancreas that is associated with obesity and metabolic syndrome can be used for estimating the clinical course of patients with COVID-19. Fat accumulation was determined by comparing the pancreas and spleen using computed tomography tools. In our study with 396 COVID-19 patients, the patients with fatty pancreas clearly seemed to have more severe clinical findings. Similarly, the rates of the requirement for hospitalization/intensive care unit and length of hospitalization were higher than in patients without fatty pancreas. The median length of hospitalization was 9 days in patients with fatty pancreas and 6 days in patients without fatty pancreas. However, death rates were only slightly higher in patients with fatty pancreas.

## Tweetable abstract

In COVID-19 patients, the patients with fatty pancreas assessed using computed tomography have a more severe clinical course, higher rates of hospitalization/ICU requirement and increased length of hospitalization compared with the patients without fatty pancreas. #COVID-19 #computed tomography #fatty pancreas.

## Graphical abstract

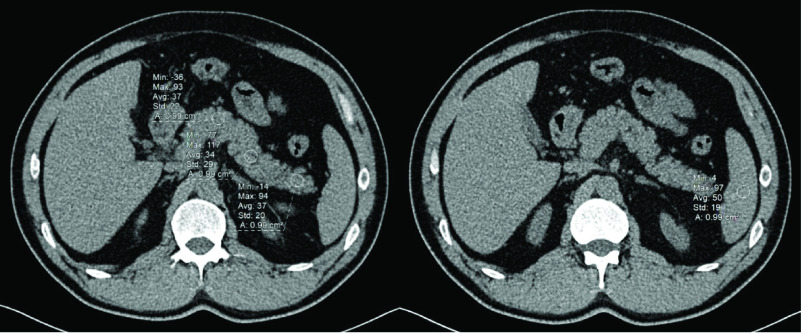

COVID-19, caused by SARS-CoV-2, first appeared in Wuhan, Hubei, China and it has rapidly spread worldwide [1]. Despite the gold standard, diagnosis remains to be a positive result of reverse transcription PCR (RT-PCR) test for the virus [2,3], chest computed tomography (CT) imaging has been playing an important role in the diagnosis and as well as in the prognosis of the disease [4–6].

Although there have been cases of asymptomatic patients and patients with mild symptoms until now, there have also been cases where the disease can progress rapidly and lead to death, especially in elderly patients and patients with accompanying chronic diseases [7]. Metabolic risk factors, such as obesity and hepatosteatosis were shown to increase the severity of the clinical findings in COVID-19 [8–10]. Hepatosteatosis is one of the pathologies which can be quantitatively evaluated on unenhanced CT. Several studies reported the relationship between hepatosteatosis and COVID-19 prognosis [8,11–13]. Similar to hepatosteatosis, pancreatic steatosis (PS) can be also assessed on CT, and its association with several diseases, such as metabolic syndrome and pancreatic cancer was reported in the literature [14,15]. To the best of our knowledge, the relationship between PS and the prognosis of COVID-19 has never been published.

We hypothesized that PS could correlate with the severity of clinical findings, the requirements for hospitalization or intensive care unit (ICU) and also the mortality of patients with COVID-19. The purpose of this study was to investigate the relationship between PS assessed with CT and the prognosis of COVID-19.

## Materials & methods

### Study population

We searched the patient records of 542 patients with a positive RT-PCR test result for SARS-CoV-2, who underwent chest CT using a 64-slice CT scanner (Siemens, Somatom Definition Flash, Erlangen, Germany) between March 2020 and December 2020 at a single center. The patients, who were under the age of 18 years (n = 5), with pancreatic mass (n = 3) or splenectomy (n = 1), who underwent contrast-enhanced chest CT (n = 16), in whom CT scans did not cover pancreas (n = 114) and in those who have significant artifacts (n = 7) were excluded, leaving 396 patients (227 males and 169 females) with a mean age of 52.50 ± 16.86 years.

The patient demographics, accompanying chronic diseases (diabetes mellitus, hypertension, coronary artery disease and cerebrovascular disease), the symptoms, the presence of positive findings on chest CT, respiratory rate and levels of oxygen saturation were noted throughout the research process. Additionally, the requirement for hospitalization or ICU, the length of stay in the hospital, need for intubation and mortality rates were logged as well. The patients were clinically classified under four groups according to mild, moderate, severe and critical findings [16]. Here, mild findings refer to mild clinical findings without positive findings on CT. Moderate findings refer to findings including fever and respiratory tract with positive findings on CT. Severe findings refer to respiratory distress with a respiratory rate ≥30-times/min and an oxygen saturation ≤93% in rest. Last, critical findings refer to respiratory failure requiring mechanical ventilation and failures of other organs requiring ICU care [16].

### CT image acquisition

All CT scans were obtained using a 64-slice scanner without using contrast media. The patients were in a supine position, and the images were obtained under breath-hold condition. The entire thoracic cavity and upper abdominal region were scanned. The CT protocol was as follows: helical scanning mode; tube voltage: 120 kV; tube current-time product: 50–350 mAs; pitch: 1.2 and 1.375; matrix: 512 × 512; reconstructed in lung and soft tissue windows; reconstructed slice thickness: 1.00 mm.

### Assessment of pancreatic steatosis

**Figure 1.**
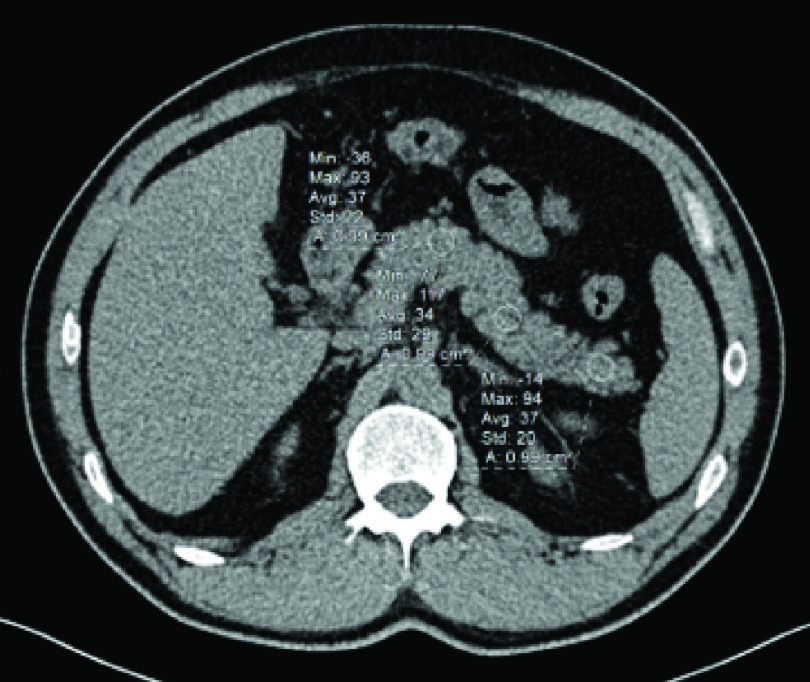
Unenhanced axial computed tomography image of a 43-year-old male patient with COVID-19. Five different ROIs (using a circular ROI of 1 cm^2^) were drawn over five anatomical parts of the pancreas to assess the attenuation value of the organ by taking the average of the 5 HU values collected from the ROIs. Shown here are the measurements from the neck, body and tail of the pancreas (37, 34 and 37 HU, respectively). The average attenuation value of the pancreas was 35 HU in this patient. HU: Hounsfield unit; ROI: Region of interest.

**Figure 2.**
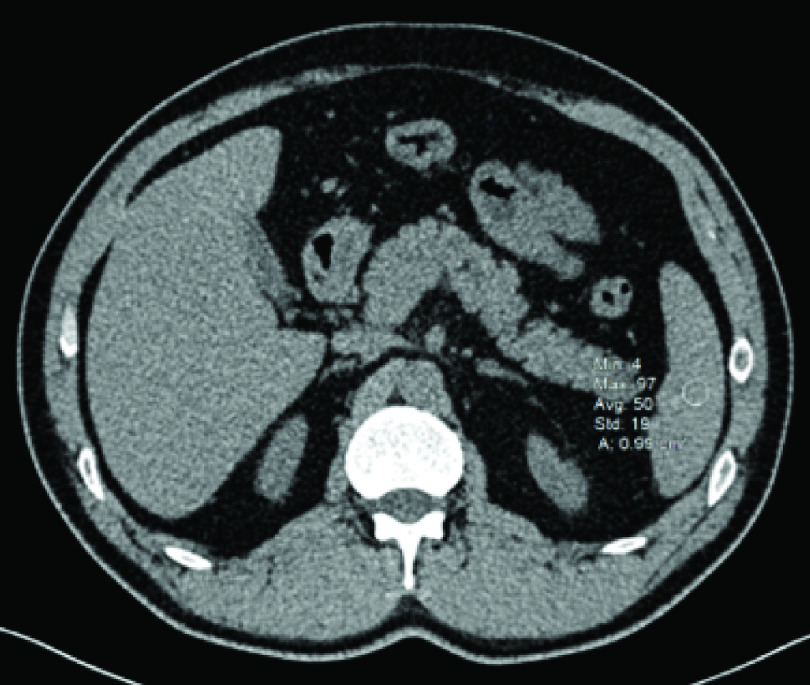
Axial computed tomography image of the same patient. Three ROIs (using a circular ROI of 1 cm^2^) were drawn from the upper, middle and lower parts of the spleen, to assess the attenuation value of the organ by taking the average of the three HU values collected from the ROIs. Shown here is the measurement from the lower a third of the spleen (50 HU). The average attenuation value was 51 HU in the patient. By dividing the attenuation value of the pancreas with the attenuation value of the spleen, a pancreas-to-spleen attenuation ratio of 0.68 was found, which was smaller than 0.70 (cut-off ratio of pancreas-to-spleen attenuation for pancreatic steatosis), confirming the diagnosis of pancreatic steatosis in this patient. HU: Hounsfield unit; ROI: Region of interest.

CT images were evaluated by two radiologists (S Guneyli and H Dogan with 11 and 2 years of experience, respectively) experienced in thoracic and abdominal radiology, who were blinded to the clinical and laboratory results of the patients, using a dedicated workstation based on consensus. Hounsfield unit (HU) of fat tissue ranges between -150 and -30 HU on unenhanced CT [14]. Both the pancreas-to-spleen (P/S) attenuation ratio and the pancreas minus spleen (P-S) attenuation difference significantly correlated with PS, proven histopathologically [17–19]. Five different regions of interest (ROIs) were drawn over the uncinate process, the head, neck, body and tail of the pancreas (Figure 1), while three ROIs were drawn from the upper, middle and lower parts of spleen (Figure 2) [15]. In both organs, a circular ROI of 1 cm^2^ was drawn [14]. To ensure reproducibility of the measurements, the ROIs were drawn by avoiding vessels and parenchymal calcifications. The average of the ROIs was calculated, and then the P/S attenuation ratio was determined, by dividing the attenuation value of the pancreas with the attenuation value of the spleen. PS was considered present in a patient when the P/S attenuation ratio was found to be <0.70 [18].

### Statistical analysis

The SPSS17.0 software (IL, USA) was used for statistical analysis. PS was compared with the severity of clinical findings, comorbidities and the requirement for hospitalization using the chi-square test. In hospitalized patients, PS was compared with the length of stay in the hospital using the Mann–Whitney U test, while it was compared with the requirement for ICU, intubation and mortality using the chi-square test. Age was expressed as mean ± standard deviation, and the length of stay in the hospital was expressed as median and range. A p value < 0.05 was considered statistically significant.

## Results

Among a total of 396 patients, 126 (31.8%) had PS, while 270 (68.2%) did not. The comparisons of the patients with and without PS according to their clinical groups and their requirement for hospitalization are presented in Table 1.

**Table 1.** Comparisons of the patients with and without pancreatic steatosis according to their clinical groups and their requirement for hospitalization among a total of 396 patients.

	PS + (n = 126)	PS – (n = 270)	p-value
Clinically mild group	9 (7.1%)	62 (22.9%)	<0.001^†^
Clinically moderate group	45 (35.7%)	159 (58.8%)	<0.001^†^
Clinically severe group	35 (27.7%)	29 (10.7%)	<0.001^†^
Clinically critical group	37 (29.3%)	20 (7.4%)	<0.001^†^
Hospitalization requirement	97 (76.9%)	104 (38.5%)	<0.001^†^

† Statistically significant values are p < 0.05.

PS: Pancreatic steatosis.

The number of patients in the four clinical groups classified from mild to critical was as follows: 71, 204, 64 and 57, respectively. There was a significant correlation between PS and the severity of clinical findings of COVID-19 (p < 0.001). Only 9 out of 126 (7.1%) patients with PS were classified in clinically mild group, whereas only 20 out of 270 (7.4%) patients without PS were classified under the clinically critical group. The rates of the patients with PS in the four clinical groups from mild to critical were respectively as follows: 12.7, 22.1, 54.7 and 64.9%. PS was found to be significantly correlated with the requirement for hospitalization in COVID-19 (p < 0.001), and the hospitalization rates in patients with and without PS were 76.9 and 38.5%, respectively. Diabetes mellitus (p = 0.810), hypertension (p = 0.057), coronary artery disease (p = 0.221) and cerebrovascular disease (p = 0.738) did not show any significant correlation with PS.

There were 201 (50.8%) in-patients and 195 (49.2%) out-patients. Out of the 201 hospitalized patients, 97 (48.2%) had PS, while 104 (51.8%) did not. Among the hospitalized patients, the comparisons of the patients with and without PS according to the four aforementioned prognostic parameters are presented in Table 2. The length of stay in the hospital among patients with PS (p = 0.002) and the requirement for ICU (p = 0.003) were higher than those without PS. The median values for the length of hospitalization in patients with PS and in those without PS were 9 (range: 2–48 days) and 6 (range: 1–92 days) days, respectively. Although, there was not any significance for the need for intubation (p = 0.104), mortality rates (p = 0.163) between the clinical groups were found to be higher in the patients with PS compared with those without PS.

**Table 2.** Comparisons of the patients with and without pancreatic steatosis according to the length of their hospitalization, their requirement for intensive care unit, their need for intubation and their mortality rates among a total of 201 hospitalized patients.

	PS + (n = 97)	PS – (n = 104)	p-value
Length of hospitalization, days (range)	9 (2–48)	6 (1–92)	0.002^†^
Requirement for ICU	37 (38.1%)	20 (19.2%)	0.003^†^
Need for intubation	26 (26.8%)	18 (17.3%)	0.104
Mortality rates	18 (18.6%)	12 (11.5%)	0.163

† Statistically significant values are p < 0.05.

ICU: Intensive care unit, PS: Pancreatic steatosis.

## Discussion

This study shows that PS is correlated with the prognostic factors, including the severity of clinical findings, the requirements for hospitalization or ICU and length of hospitalization in COVID-19 patients; while comorbidities, the need for intubation and mortality rates did not correlate with PS.

There is an increase in the research of PS in the literature recently, which can be attributed to the increasing prevalence of obesity and metabolic syndrome among patients worldwide. The metabolic syndrome causes fatty infiltration in many organs, including the heart, liver and subcutaneous tissues which can lead to chronic inflammation, fibrosis and organ dysfunction [20]. Chronic lipogenic and glucogenic inflammation are thought to be associated with PS. It was reported that PS can lead to acute pancreatitis, diabetes mellitus and pancreatic malignancy [15,21].

CT is the most commonly used imaging modality in evaluating PS. Kim *et al.* reported that the histologic pancreatic fat fraction significantly correlated with the P-S attenuation difference assessed on CT, and the sensitivity and specificity of PS were stated as 79.3 and 42.4%, respectively [17]. In the study of Limanond *et al.*, unenhanced CT was used to investigate the correlation between PS and metabolic syndrome, and they considered PS to be present when there is a P-S attenuation difference that is less than -5 HU [22]. Unlike this study, we used the P/S attenuation ratio in the assessment of PS. In order to obtain more accurate density values, five ROIs were used instead of three or fewer ROIs. Additionally, obtaining these five ROIs from five anatomical parts of the pancreas can lead to acquiring more accurate density values on CT compared with when the ROIs are obtained randomly.

Obesity and hepatosteatosis were reported in the literature as potential risk factors in patients with COVID-19 [8,23]. Similar to hepatosteatosis, PS is associated with obesity and metabolic syndrome. This association can point to a potential relationship between PS and the clinical severity of COVID-19. Furthermore, compared with the evaluation of hepatosteatosis on chest CT, evaluating PS could prove to be a more efficient and reliable method. This is because the pancreas is an organ that is covered mostly on chest CT while the liver is not covered entirely, and because the fatty infiltration of pancreas is relatively more diffuse compared with the fatty changes of the liver on CT. In our study, the rates of the patients with PS were higher in patients who were in the clinically critical group and in patients requiring hospitalization. As the clinical group moved from mild to critical, in other words, as the clinical course worsened, the rate of patients with PS gradually increased, especially in the clinically severe and critical groups. However, the accompanying chronic diseases were not found to be significant when they were compared between the groups.

Among the 201 hospitalized patients, 97 patients had PS, whose length of hospitalization and rates of ICU requirement were higher than those without PS. Apart from the impact of the clinically severe disease and its complications on the prognosis, if the length of hospital or ICU stay increases, the patients may develop several hospital-related complications, such as nosocomial infections and iatrogenic complications. There were not any significant results gathered from comparing the need for intubation and the mortality rates between patients with and without PS. This can be explained by the relatively low numbers of patients requiring intubation and low mortality rates.

This study has several limitations. First, the study had a retrospective design and was conducted in a single center. Second, the baseline data for the patients was absent. Third, the degree of PS severity was not considered as a parameter in our study, and the assessment of PS, by using a cut-off ratio on CT seemed to be less specific compared with that which was obtained from histopathologic specimens. Additionally, some patients did not have a homogeneous fat distribution in their pancreas. However, the use of five ROIs obtained from five anatomical parts of the pancreas, which is more than the average amount of ROIs used in similar studies from the literature, is thought to minimize this limitation. Finally, the HU values of the pancreas were obtained with the consensus of two radiologists instead of having two separate assessments.

## Conclusion

PS can be effectively demonstrated using unenhanced chest CT in patients with COVID-19, which can readily be performed in most of the COVID-19 patients. It can be used as a prognostic factor in COVID-19, since it is in correlation with the severity of clinical findings in COVID-19, the requirement for hospitalization or ICU, and the length of hospitalization in COVID-19 cases. Although the need for intubation and the mortality rates were not found to be of significance, they were observed to be higher in patients with PS. Future studies with larger sample sizes can demonstrate the possible associations between PS and other prognostic factors. With the further investigation of PS and the degree of PS severity in future studies, the clinical importance and the use as a prognostic factor of this phenomenon in COVID-19 can be shown to be even more crucial.

**Summary points**

- Pancreatic steatosis (PS) that is associated with metabolic syndrome can be also used for estimating the prognosis in patients with COVID-19.
- Both the pancreas-to-spleen attenuation ratio and the pancreas-minus-spleen attenuation difference were reported to be in correlation with PS, which were proven via histopathologic confirmation.
- The mean age of 396 patients (227 males and 169 females) was 52.50 ± 16.86 years, and PS was found to be present in 126 of 396 (31.8%) patients.
- There was a strong correlation between PS and severity of clinical findings in COVID-19, and the rates of the patients with PS gradually increased from the clinically mild to the clinically critical groups, in the order of 12.7, 22.1, 54.7 and 64.9%, respectively.
- PS was in correlation with the requirement for hospitalization or intensive care unit, and with the length of hospitalization.
- The median values of the length of hospitalization in patients with PS and without PS were 9 (range: 2–48 days) and 6 (range: 1–92 days) days, respectively.
- Although the need for intubation and mortality rates among hospitalized COVID-19 patients with PS were higher than those without PS, these parameters did not reach a significant level.

## References

[1] Han R, Huang L, Jiang H, Dong J, Peng H, Zhang D. Early clinical and CT manifestations of coronavirus disease 2019 (COVID-19) pneumonia. AJR Am. J. Roentgenol. 215(2), 338–343 (2020). 32181672 10.2214/AJR.20.22961

[2] Corman VM, Landt O, Kaiser M Detection of 2019 novel coronavirus (2019-nCoV) by real-time RT-PCR. Euro. Surveill. 25(3), 3–11 (2020). 10.2807/1560-7917.ES.2020.25.3.2000045 PMC6988269 31992387

[3] Rubin EJ, Baden LR, Morrissey S, Campion EW. Medical journals and the 2019-nCoV outbreak. N. Engl. J. Med. 382(9), 866 (2020). 31986242 10.1056/NEJMe2001329

[4] Chung M, Bernheim A, Mei X CT imaging deatures of 2019 novel coronavirus (2019-nCoV). Radiology 295(1), 202–207 (2020). 32017661 10.1148/radiol.2020200230 PMC7194022

[5] Pan Y, Guan H, Zhou S Initial CT findings and temporal changes in patients with the novel coronavirus pneumonia (2019-nCoV): a study of 63 patients in Wuhan, China. Eur. Radiol. 30(6), 3306–3309 (2020). 32055945 10.1007/s00330-020-06731-x PMC7087663

[6] Güneyli S, Atçeken Z, Doğan H, Altınmakas E, Atasoy KÇ. Radiological approach to COVID-19 pneumonia with an emphasis on chest CT. Diagn. Interv. Radiol. 26(4), 323–332 (2020). 32352917 10.5152/dir.2020.20260 PMC7360081

[7] Zhou F, Yu T, Du R Clinical course and risk factors for mortality of adult inpatients with COVID-19 in Wuhan, China: a retrospective cohort study. Lancet 395(10229), 1054–1062 (2020). 32171076 10.1016/S0140-6736(20)30566-3 PMC7270627

[8] Çoraplı M, Çil E, Oktay C, Kaçmaz H, Çoraplı G, Bulut HT. Role of hepatosteatosis in the prognosis of COVID 19 disease. Clin. Imaging 80, 1–5 (2021). 34214871 10.1016/j.clinimag.2021.06.034 PMC8234248

[9] Guo W, Li M, Dong Y Diabetes is a risk factor for the progression and prognosis of COVID-19. Diabetes. Metab. Res. Rev. (2020) (Epub ahead of print). 10.1002/dmrr.3319 PMC7228407 32233013

[10] Cai Q, Chen F, Wang T Obesity and COVID-19 severity in a designated hospital in Shenzhen, China. Diabetes Care 43(7), 1392–1398 (2020). 32409502 10.2337/dc20-0576

[11] Gao F, Zheng KI, Wang XB Metabolic associated fatty liver disease increases coronavirus disease 2019 disease severity in nondiabetic patients. J. Gastroenterol. Hepatol. 36(1), 204–207 (2021). 32436622 10.1111/jgh.15112 PMC7280625

[12] Medeiros AK, Barbisan CC, Cruz IR Higher frequency of hepatic steatosis at CT among COVID-19-positive patients. Abdom. Radiol. 45(9), 2748–2754 (2020). 10.1007/s00261-020-02648-7 PMC7368629 32683613

[13] Palomar-Lever A, Barraza G, Galicia-Alba J Hepatic steatosis as an independent risk factor for severe disease in patients with COVID-19: a computed tomography study. JGH. Open 4(6), 1102–1107 (2020). 10.1002/jgh3.12395 PMC7436487 32838045

[14] Hoogenboom SA, Bolan CW, Chuprin A Pancreatic steatosis on computed tomography is an early imaging feature of pre-diagnostic pancreatic cancer: a preliminary study in overweight patients. Pancreatology 21(2), 428–433 (2021). 33485792 10.1016/j.pan.2021.01.003

[15] Lee JS, Kim SH, Jun DW Clinical implications of fatty pancreas: correlations between fatty pancreas and metabolic syndrome. World. J. Gastroenterol. 15(15), 1869–1875 (2009). 19370785 10.3748/wjg.15.1869 PMC2670415

[16] Li K, Fang Y, Li W CT image visual quantitative evaluation and clinical classification of coronavirus disease (COVID-19). Eur. Radiol. 30(8), 4407–4416 (2020). 32215691 10.1007/s00330-020-06817-6 PMC7095246

[17] Kim SY, Kim H, Cho JY Quantitative assessment of pancreatic fat by using unenhanced CT: pathologic correlation and clinical implications. Radiology 271(1), 104–112 (2014). 24475851 10.1148/radiol.13122883

[18] Fukuda Y, Yamada D, Eguchi H CT density in the pancreas is a promising imaging predictor for pancreatic ductal adenocarcinoma. Ann. Surg. Oncol. 24(9), 2762–2769 (2017). 28634666 10.1245/s10434-017-5914-3

[19] Navina S, Acharya C, DeLany JP Lipotoxicity causes multisystem organ failure and exacerbates acute pancreatitis in obesity. Sci. Transl. Med. 3(107), 107ra110 (2011). 10.1126/scitranslmed.3002573 PMC3321362 22049070

[20] Tran V, De Silva TM, Sobey CG The vascular consequences of metabolic syndrome: rodent models, endothelial dysfunction, and current therapies. Front. Pharmacol. 11, 148 (2020). 32194403 10.3389/fphar.2020.00148 PMC7064630

[21] Guglielmi V, Sbraccia P. Type 2 diabetes: does pancreatic fat really matter? Diabetes. Metab. Res. Rev. 34(2), e2955 (2018). 10.1002/dmrr.2955 28984071

[22] Limanond P, Raman SS, Lassman C Macrovesicular hepatic steatosis in living related liver donors: correlation between CT and histologic findings. Radiology 230(1), 276–280 (2004). 14695401 10.1148/radiol.2301021176

[23] Sachdeva S, Khandait H, Kopel J, Aloysius MM, Desai R, Goyal H. NAFLD and COVID-19: a pooled analysis. SN. Compr. Clin. Med. 6, 1–4 (2020). 10.1007/s42399-020-00631-3 PMC7646222 33173850

